# The Relative Importance of “Cooperative Context” and Kinship in Structuring Cooperative Behavior: A Comparative Study of Saami Reindeer Herders

**DOI:** 10.1007/s12110-021-09416-6

**Published:** 2021-10-20

**Authors:** Guro Lovise Hole Fisktjønmo, Marius Warg Næss, Bård-Jørgen Bårdsen

**Affiliations:** 1 High North Department, Fram Centre, Norwegian Institute for Cultural Heritage Research, 9296 Tromsø, Norway; 2 Arctic Ecology Department, Fram Centre, Norwegian Institute for Nature Research, 9296 Tromsø, Norway

**Keywords:** Collaboration, Nomadic pastoralism, Kin selection, Reciprocal altruism, Social groups, Reindeer herding

## Abstract

Kin relations have a strong theoretical and empirical basis for explaining cooperative behavior. Nevertheless, there is growing recognition that context—the cooperative environment of an individual—also shapes the willingness of individuals to cooperate. For nomadic pastoralists in Norway, cooperation among both kin and non-kin is an essential predictor for success. The northern parts of the country are characterized by a history of herder-herder competition exacerbating between-herder conflict, lack of trust, and subsequent coordination problems. In contrast, because of a history of herder-farmer competition, southern Norway is characterized by high levels of between-herder coordination and trust. This comparative study investigates the relative importance of “cooperative context” and kinship in structuring cooperative behavior using an experimental gift game. The main findings from this study were that in the South, a high level of cooperation around an individual pushes gifts to be distributed evenly among other herders. Nevertheless, kinship matters, since close kin give and receive larger gifts. In contrast, kinship seems to be the main factor affecting gift distribution in the North. Herders in the North are also concerned with distributing gifts equally, albeit limiting them to close kin: the level of intragroup cooperation drives gifts to be distributed evenly among other closely related herders. The observed regional contrasts in cooperative decisions fit with the different historical levels of conflict and trust in the two regions: whereas herders in the South are affected by both cooperative context and kinship, kinship seems to be the main determinant of cooperation in the North.

**Supplementary Information:**

The online version contains supplementary material available at 10.1007/s12110-021-09416-6.

In anthropology, the evolution of cooperation is often framed with respect to forager societies. The underlying hypothesis is that they have a close link to our evolutionary past—living in an evolutionarily relevant context (Apicella & Silk, 2019; Apicella et al., 2014; Chaudhary et al., 2015; Hill et al., 2014; Smith et al., 2018). Far less is known about cooperation among nomadic pastoralists even though they cooperate extensively in so-called herding groups (Næss, 2012). A case in point is the Saami *siida* system found in Fennoscandia: a socioeconomic group whose members are united by kinship, live close together, and pursue a common economic goal, that of successfully herding reindeer. Moreover, it is relatively small and flexible: it changes in size and composition throughout the season, and members are relatively free to change group affiliation. Being based on kinship—formed around a core sibling group—the siida allows members to maintain face-to-face communication, monitor each other, and punish individuals who break rules. These are characteristics that to a large degree favor the maintenance of cooperation as well as deter free-riding tactics (e.g., Alvard, 2003; Griffin & West, 2002). Furthermore, being informally led by a wealthy and skillful person, although decision-making is mostly consensual, the herding groups are mildly hierarchical (i.e., characterized by relative egalitarianism; Næss, 2020; Næss et al., 2021). The Saami siida system is thus a well-suited area for extending our understanding of nomadic pastoral cooperation.

## The Evolutionary Aspect of Cooperation

Cooperation can be defined as a collective action wherein at least two individuals interact or coordinate actions to achieve a mutual benefit (Smith, 2003). Thus, cooperation will evolve if the fitness benefit from cooperation outweighs the cost (West et al., 2007). From an evolutionary perspective, explanations of cooperative behavior are broadly classified into two categories: direct or indirect fitness benefits (West et al., 2011). A direct benefit occurs if the cooperative behavior benefits both the actor and the recipient (West et al., 2007). Cooperative hunting is likely to be maintained when coordinated action increases individual hunting success, prey encounter rates, or harvest size, or reduces the costs of search and pursuit—in short, when it leads to an increase in per capita foraging return rates (Alvard & Nolin, 2002; Kaplan et al., 1990; Smith, 1981). Among whale hunters in Indonesia, cooperative whale hunting resulted in greater per capita returns than solitary fishing (Alvard & Nolin, 2002). Thus, cooperation makes it possible to obtain benefits that are not easily obtained by individuals acting alone (Axelrod, 1984).

Indirect fitness benefits accrue, on the other hand, when an individual can benefit by cooperating with other individuals who share the same cooperative gene (West et al., 2007): in other words, individual benefits are acquired indirectly through kin relations. Numerous studies have shown that cooperative behavior among kin is more extensive than what is predicted to occur by chance alone (Gibson & Mace, 2005; Kramer, 2005). Among the Martu in Australia, Bliege Bird et al. (2012) found that hunters cooperate more often with kin than with non-kin. More generally, Kaplan and Gurven (2005) argue that among foragers, meat distribution is biased toward close kin living in other families at the expense of distant kin and unrelated families. Among the Aché in Paraguay, for example, there is a strong kin bias toward between-family sharing in the settlements, but not in forest camps (Gurven et al., 2002). Interhousehold food sharing among the Dolgan and Nganasan in Siberia shows a similar trend: food transfers increase in frequency when the degree of relatedness increases (Ziker & Schnegg, 2005; Ziker et al., 2016). Among pastoralists, previous studies on Saami reindeer husbandry in Norway have found that kinship influences slaughter strategies (Næss et al., 2012), herd size (Næss et al., 2010), and the probability of gift-giving (Thomas et al., 2015). In sum, individuals in small-scale societies preferentially aid close kin over more distant kin and non-kin (Allen-Arave et al., 2008; Borgerhoff Mulder, 2007).

## Interdependent Decision-Making

Although kin selection is an essential mechanism for cooperative behavior, studies of present-day foraging societies show that their social structure is characterized by high mobility and residential mixing, consisting of a substantial number of unrelated individuals (Hill et al., 2011). In a study of 32 forager societies, Hill et al. (2011) found that primary kin generally made up less than 10% of a band. In a study of six Agta camps and three BaYaka camps (Congo-Brazzaville), Migliano et al. (2017) found that friendship was essential: both groups had between one and four unrelated “close friends” with whom they interact as frequently as they do with close kin. Moreover, among whale hunters in Indonesia, lineage membership rather than genetic kinship determined hunting group formation (Alvard, 2003; see also Allen-Arave et al., 2008).

Thus, not only kin selection facilitates cooperation. For example, an individual’s best choice of action also depends on the action that person expects other individuals to take (Schelling, 1980). Gurven (2006) found evidence of significant contingency in food exchange for both Aché and Hiwi (Colombia). Among the Hiwi there is a strong contingency concerning sharing of meat and fish: for every kilogram of meat and other foods given to another family, the giver will receive 0.69 kg of meat and 0.08 kg of other food, in return, on a later occasion (Gurven, 2006). Among the Agta in the Philippines, Smith et al. (2019) found that sharing was based on the recipients’ level of need, kin relation, and reciprocity. The latter implies that they share with individuals who have shared with them previously. Pastoralist households in Namibia transfer food to others on demand through a norm of sharing (Schnegg, 2015). Despite this sharing norm, reciprocal relationships emerged within spatial clusters around households, especially for high-value goods, and did not appear to be influenced by kinship (Schnegg, 2015). Moreover, Nolin (2010) found among Lamalera whale hunters in Indonesia that reciprocal altruism is the primary motivation for food sharing whereas kinship and distance appear to be critical partner-choice criteria.

As opposed to what would be expected if individuals choose cooperative partners based on a stable level of cooperation, a study of Hadza hunter-gatherers in Tanzania found no evidence that the cooperative behavior of individuals persists over time (Smith et al., 2018). Instead, Smith et al. (2018) found that one of the strongest predictors of an individual’s willingness to cooperate was the social context. In a public goods game, they found that for each additional honey stick contributed by camp members, the giver contributed, on average, another half-stick of honey (Smith et al., 2018). A similar pattern has been found in Swiss school classes. Investigating the importance of social learning and culture for the emergence of cooperation, Ehlert et al. (2020) found that students embedded in cooperative friendship environments became, over time, substantially more cooperative than their peers in comparable, less-cooperative environments. Results of previous studies of Saami reindeer husbandry have similar implications: the amount, probability, and type of animals slaughtered by an individual herder is influenced by the slaughter decisions made by neighboring herders (Næss et al., 2012). In sum, this indicates the “cooperative context” (i.e., the willingness of surrounding individuals to engage in cooperation) is a potentially important yet under-studied predictor for human cooperation.

Significantly, both theoretical work and empirical evidence suggest that kinship and reciprocity can interact synergistically to increase cooperation in small, tightly knit communities (Axelrod & Hamilton, 1981; Henrich & Muthukrishna, 2021). However, this has mainly been studied in a food-sharing context: sharing frequency increases as households become more closely related and as the frequency of reciprocal food transfers increases (Koster, 2011; Ziker & Schnegg, 2005). Specifically, among the Dolgan and Nganasan in Siberia, Ziker et al. (2016) investigated how food sharing was affected by hunting skill, reciprocity, and kinship and found interactions between hunting skills and reciprocity and between kinship and reciprocity. Less evidence is available concerning cooperation among nomadic pastoralists. In Norway, kinship has a mediating effect on cooperative production: high levels of relatedness coupled with high access to labor had an increasingly positive effect on herd size (Næss et al., 2010). Thus, investigating the interactions between kinship and the cooperative context will extend our understanding of pastoral cooperation.

## Predictions

Experimental games in which participants can distribute money or other valuables have proven to be a fruitful method for measuring cooperative behavior (Henrich et al., 2001, 2006, 2010; Smith et al., 2018; Thomas et al., 2015, 2016). Previous studies of gift-giving behavior among the Saami found that the strongest predictor for gifting was membership in the same siida (Thomas et al., 2015). Giving gifts entails two different—yet interlinked—decisions: (1) to whom one wants to give a gift and (2) how much to give. Thomas et al. (2015) found that the *probability* of giving a gift increased with membership in the same siida, but it is not apparent that the same should be the case for deciding the size of the gift. In a probabilistic context, gifts of 5 or 100 L of petrol are equal. Sizes of gifts are, however, not equal. In effect, although siida membership might be an essential factor when deciding who should receive gifts, the size of the gifts might be influenced by kinship. This is in line with Essock-Vitale and McGuire (1980), who argue that kin will be given more support than non-kin, and close kin will receive the most; in effect, large gifts might be more likely both to be given to and to come from close kin. Similarly, Mysterud et al. (2006) found that kinship was an important predictor for gift-giving at Christmas among Norwegian students: more money was spent on gifts for close kin (i.e., close kin both received and gave more valuable gifts to each other). Using an experimental gift game (Apicella et al., 2012; Thomas et al., 2015), this paper thus aims to investigate how the size of the gifts herders give and receive is influenced by the cooperative context and kinship. Pertinently, since both theoretical work and empirical evidence suggest that kinship and reciprocity can interact (see above), we are also interested in assessing how the interaction between kinship and the cooperative context influences the size of the gifts herders give and receive.

**Fig. 1:**
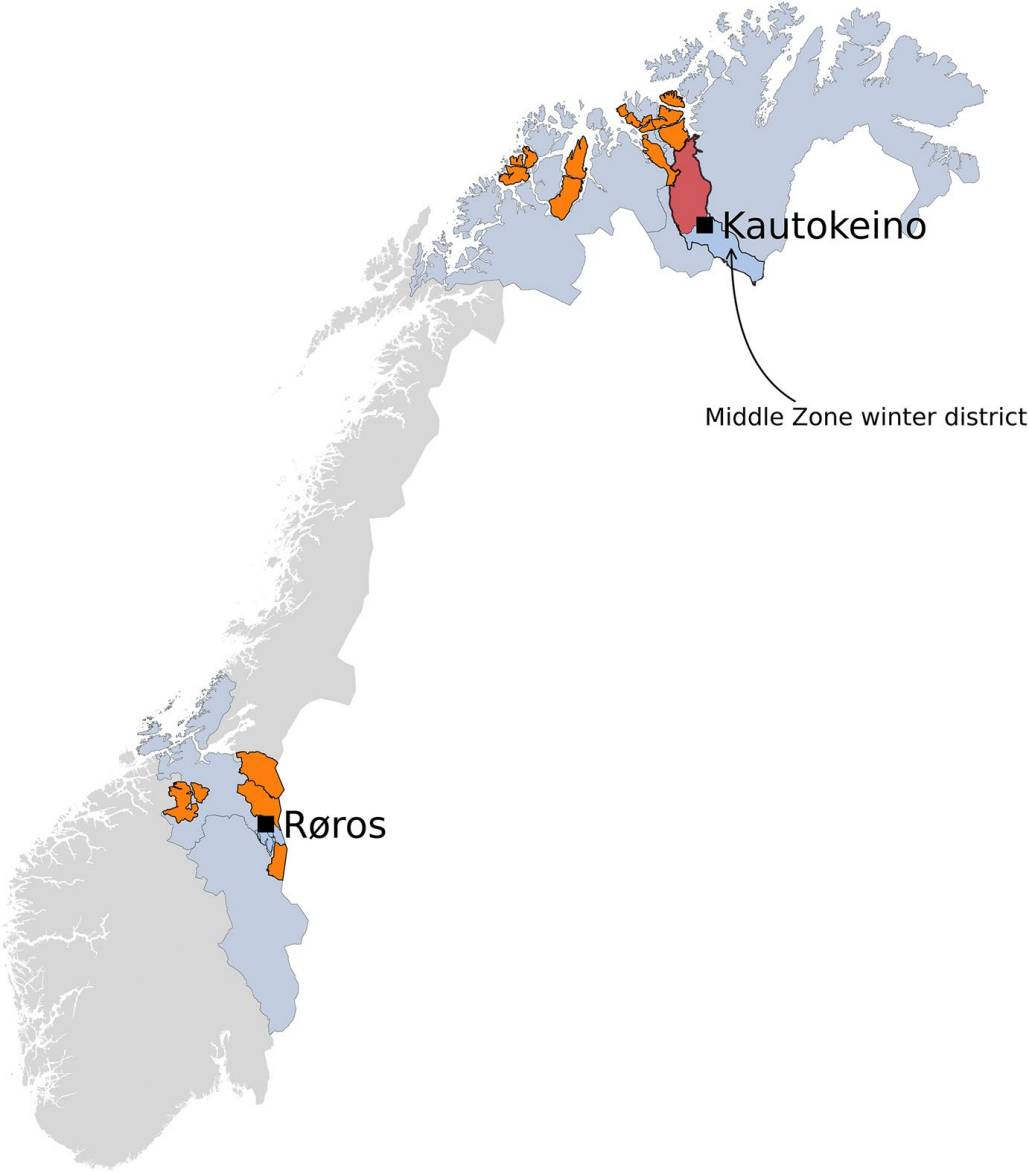
Map of the study area in Norway. Kautokeino is in Troms and Finnmark County (marked with light blue) in the North. Røros is in Sør-Trøndelag and Hedmark County (marked with light blue) in the South. Reindeer districts are marked with orange, red, and blue. Map created in Python 3.8.10 (https://www.python.org/) with background map from GADM (https://gadm.org/maps/NOR.html and official reindeer districts from the Norwegian Institute of Bioeconomy Research’s Kilden (https://kart8.nibio.no/nedlasting/dashboard)

Importantly, Riseth and Vatn (2009; see also Næss & Bårdsen, 2015) argue that reindeer herders in the counties of Finnmark and Troms (hereafter “North”) are faced with more problems concerning coordination than herders in Sør-Trøndelag/Hedmark (hereafter “South”; Fig. 1). Næss (2020) has argued that this stems from a historical difference: whereas herders in the North have mainly competed against each other, herders in the South have mainly competed against an expanding farming sector. In the North, a history of competition between herders has exacerbated conflict and lack of trust (for details, see Næss, 2020). In contrast, herders in the South have had to deal with an expanding agricultural sector, resulting in intense competition for land. Continued pressure from farmers required the herders to present a common front vis-a-vis the farming community (Holand, 2003). Thus, in the South, a history of herder-farmer conflicts has increased herder coordination and trust (Næss, 2020). Consequently, we expect that the effect of kinship and level of intragroup cooperation differs regionally, with kinship being more critical in the North and the cooperative context more critical in the South. This gives rise to the following regional predictions:

## North

1. Kinship is a better predictor of cooperation in the North compared with the South. Thus, we expect kinship to be a positive predictor for the size of gifts both given and received. In effect, if kinship is the primary mechanism for cooperation, we expect that herders will give fewer but larger gifts to close kin.
2. The interaction between kinship and cooperative context is expected to be determined more by kinship in the North, meaning that kinship weakens the effect of the cooperative context in the North. In effect, herders give and receive fewer, but larger, gifts in the North than in the South.

## South

3. Cooperation around a given herder is more critical in the South than the North. The level of cooperation around a herder (i.e., the “cooperative context”) is thus expected to have a negative effect on the size of gifts given and received. This because when a larger number of gifts is distributed in a siida, herders must divide the gift among more herders.
4. In the South, the interaction between kinship and the cooperative context is expected to be determined more by the cooperative context, meaning that the cooperative context weakens the effect of kinship. In effect, herders give and receive more, but smaller, gifts in the South than in the North.

## Methods

### Reindeer Husbandry in Norway

Reindeer husbandry, the cornerstone of the indigenous Saami culture (Bostedt, 2001), developed as a pastoral economy at least 400 years ago and probably evolved from a hunting culture based on wild reindeer (Paine, 1994; Riseth & Vatn, 2009; see Bergstrøm, 2005; Bjørklund, 1990; Bostedt, 2001; Hansen & Olsen, 2004 for other estimates). From a national point of view, Saami reindeer husbandry is a relatively small industry, consisting of 538 siida *shares* and 3307 affiliated people. Nevertheless, the Saami reindeer husbandry is vital from a local and Saami point of view in terms of both economy and culture. Moreover, around 40% of Norway’s landmass is used by reindeer herders (Næss & Bårdsen, 2015).

Saami reindeer herders’ social organization has three layers. The basic unit is the *siida-share,* a license granted by the government entitling the owner to manage a herd of reindeer within a designated area. One or more license owners belong to a *siida* (North) or *sijte* (South; but the official designation is *siida*). The siida is a cooperative herding group composed of independent households and traditionally organized around kinship (siidas can also include non-kin); there are 99 summer siidas and 150 winter siidas in Norway (Norwegian Agriculture Agency, 2018). Siidas are grouped into *districts—*formal administrative units defined by the government (Næss & Bårdsen, 2013, 2015, and see below). Although siidas and districts are formally different, in many instances, the siida and the summer district represent an equivalent unit: Hausner et al. (2012) report that 39 of the 44 summer districts in the North consist of only one siida, while the remaining districts have two or three siidas.^1^ The four summer districts in the South consist of only one siida each; one winter district is shared between two summer districts (Norwegian Agriculture Agency, 2018). For more information, see Næss (2020).

### Regional Differences

The most evident regional difference between North and South concerns reindeer abundance (Riseth, 2003; Tveraa et al., 2007).^2^ Between the 1960s and 1990s, the South was characterized by stable reindeer populations (Riseth & Vatn, 2009). Furthermore, whereas abundance in the North has continued to increase until recently, the reindeer population seems to have decreased in the South (Næss & Bårdsen, 2015). Additionally, herders in the South tend to slaughter a larger proportion of their calves despite a relatively similar temporal trend between the two regions in net price per kilo of meat (Næss & Bårdsen, 2015).

Notwithstanding the geographical distance of approximately 1000 km, the two regions have similar access to winter pastures, which are characterized as cold, stable, and having a favorable climate for pasturage, with generally low levels of precipitation (Tveraa et al., 2007). Nevertheless, the herders in the South have access to limited winter pastures and live in a more fragmented landscape compared with the North, with its open landscape and summer pasture limitations (Riseth & Vatn, 2009). Riseth and Vatn (2009) suggest that the regional differences in abundance may be related to how herders in the two regions have handled external changes from just after the Second World War and until recently. During this time, new technologies were introduced, herders experienced increased market access, and new state policies were developed for modernizing the industry (see Næss & Bårdsen, 2013: S1).

Herders in the North, being more numerous, also have considerably more challenges when it comes to coordination of pasture use (Riseth & Vatn, 2009). Competition for pastures might thus explain why herders in the North, and not in the South, have allowed their herds to accumulate as a risk-management strategy (Næss, 2020; Næss et al., 2010). In the 1970s, the Norwegian state became more directly involved in reindeer husbandry through various subsidies and regulations. Reforms in the late 1970s and early 1980s aimed at co-management and optimization of production (Riseth & Vatn, 2009). Although the process of herd growth in the North started before these reforms, Riseth and Vatn (2009) argue that instead of curbing herd accumulation, these reforms gave an extra impetus to increasing the size of herds in the region.

In contrast, the Saami in the South participated actively in reindeer husbandry management and were in many cases themselves behind the institutional changes implemented by the authorities (Riseth & Vatn, 2009). Consequently, the new polices fitted changes the Saami themselves were advocating, turning their focus to increased meat production rather than herd accumulation (Næss, 2020).

### Study Design and Protocol

The research reported here is based on interviews undertaken with reindeer herders from the North during June and August 2016 and reindeer herders from the South during August–October 2017 and March 2018.

The study designs for the North and South were somewhat different: in the North, the explicit aim was to interview herders utilizing winter pasture areas managed as commons, namely the “Middle Zone” winter district (Fig. 1). The Middle Zone is used by herders from 12 summer districts, with three of the summer districts located in Troms County. There are 745 people (95 of whom are licensed owners) distributed among 24 winter siidas and 16 summer siidas (Norwegian Agriculture Agency, 2016). We interviewed 31 out of 95 siida-shares that use the Middle Zone winter district (see above and Table 1). The herders were distributed among five of the nine Finnmark-based summer districts and two of the three Troms-based summer districts. In the South, the explicit aim was to interview herders from all four summer districts. In the South, we interviewed 17 out of 30 siida-shares (Table 1), covering all the summer districts in the region.

**Table 1:** Descriptive statistics of the participants in the different regions

Region	Number of licenses		Mean age (SD)	
	Male	Female	Male	Female
South	14	3	54 (10.3)	58 (6.1)
North	29	2	50 (11.9)	48 (21.9)

Participants were recruited by systematically phoning all license owners, the majority of whom were either unreachable, unavailable, or unwilling to take part. A time and place for carrying out the interview was arranged with license owners willing to participate in the study. All participants provided written informed consent. The survey contained questions about the participant, personal economy, siida affiliation, and division of labor.

This study is based on three data sets. The first data set consists of general information about the owner of the reindeer herding license (*n* = 48). The second data set consists of kinship relations with other individuals in the reindeer husbandry (for details pertaining to this data set and how it was collected, see Thomas et al., 2018). The third data set consists of the results of a one-shot gift game that was conducted at the end of every interview (Table 2). The participants were given an amount of fuel to distribute among other licensed herders and decided to whom and how much they would give (for a discussion of the cultural relevance of using petrol instead of other currencies in an experimental setting involving reindeer herders, see Thomas et al., 2018). The receiver would not know the identity of the giver, and the only restriction placed on the giver was that it had to be given to another licensed herder within the region. While the game was played in terms of petrol, all herders were made aware that any winnings would be paid in NOK.

**Table 2:** Descriptive data from the gift game in the winter siidas in the North and South

Winter siida	Number of licenses	Mean *r*	Gifts			
			Total number^a^	Total amount	Mean no. per license (SD)	Mean size (SD)
**South**	30	0.12	36	1500	2.4 (2.5)	41.6 (37.3)
Saanti	9	0.12	25	700	3.6 (3.4)	28 (32.2)
Gåebrien	10	0.09	6	500	1.2 (0.2)	83.3 (25.8)
Svahken	6	0.21	1	100	1	100
Trollheimen	5	0.38	4	200	2 (0)	50 (24.5)
**North**	48	0.28	77	1050	2.7 (1.9)	13.6 (10.2)
Ánden Áilu Bardniid	5	0.38	1	35	1	35
Balsemihkkala	3	0.25	2	70	1 (0)	35 (0)
Beartašjohka	7	0.18	12	140	3 (2.2)	11.7 (8.6)
Buljo	3	0.18	5	105	1.7 (0.6)	21 (8.0)
Dommaid	4	–	3	35	3	11.9
Hánskenillasa Bárdniid	6	0.29	15	105	5 (0)	7 (0)
Ingor-Ánte Bárdniid	2	–	8	70	4 (4.2)	8.7 (10.6)
Ittunjarga	4	0.20	12	210	2 (0.6)	17.5 (6.1)
Njullosávžži	6	0.08	4	140	1 (0)	35 (0)
Oskaliid	3	0.5	4	70	2 (1.4)	17.5 (11.7)
Silvvaniid	5	0.29	11	70	5.5 (2.1)	6.4 (1.8)

Number of licenses is the total number of licenses in different regions. The amount and mean size of the gifts are given in liters. Mean *r* is the mean coefficient of relatedness (*r*) among members

^a^The table shows the total number of gifts given/received in the study. This number differs from the sample size in the statistical analyses presented in Tables 3 and 4 because everyone who has given a gift is interviewed, whereas not everyone who received a gift was interviewed, and not all participants were willing to give information about kinship. Thus, information about kinship or siida affiliation was missing for some of the gifts, leading them to be excluded from the analysis

Because of some changes in funding, the amount of fuel available for the participants to give away was higher in the South than in the North. In the North, the amount was 35 L (approximately 525 NOK or 52.34 USD); in the South the amount was 100 L of fuel (approximately 1600 NOK or 181.35 USD).^3^ After the participants had distributed the fuel, they were asked to explain why they distributed the gifts as they did. Herders in both areas were asked to give everything away to at least one other license owner but could give to as many recipients as they desired. For each gift, we recorded the anonymized ID number of the recipient, the amount given, and the reason for the gift. When there was only one participating herder in a siida, they were excluded from the statistical analyses. All gifts were given anonymously, and payments were lumped into the total amounts earned and paid via bank transfer at the end of the data collection period. Thus, no herders knew how many gifts they received or from whom they came (see also Thomas et al., 2018).

Following the Recipient Identity-Conditioned Heuristic (RICH) economic games as described by Gervais (2017), the gift game used in this study differs from standard anonymous experimental games in two ways. First, recipients’ identities were known by decision-makers: participating herders were presented with a set of known recipients. Second, gift-giving entails parallel decisions made for a selection of recipients in the herding communities—in effect, mirroring the social trade-offs in resource allocations that characterize siidas. According to Gervais (2017), identifying recipients and making a forced trade-off among them capture critical moderators of decision-making. However, experimental economic games usually incorporate some costs for the decision-maker; in the most closely related RICH game (the allocation game), participants were asked to allocate coins between themselves and others (Gervais, 2017). Thus, choosing to allocate coins to others leaves less for the decision-maker to keep. Nevertheless, although gift-giving in our procedure is cost-free, giving a gift is an important measure of friendship, or a measure of whom you want to cooperate with, and has been argued to be a valid measure of social connections (e.g., Glowacki et al., 2016). Thus, gift-giving effectively reveals information about existing social relationships, providing some insight into the patterns of cooperation among Saami reindeer herders (Thomas et al., 2018).

The data set for the analysis contains the following variables:

- *Amount*_*given*_ (response): A continuous variable denoting the size of the gift (in liters of fuel) a herder has given to another herder.
- *Amount*_*received*_ (response): A continuous variable denoting the size of the gift (in liters of fuel) a herder has received from another herder.
- *r*: a numeric variable between 0 and 1 denoting the coefficient of relatedness between the giver and the receiver. Since we limited kin relationships to second cousins (*r* < 0.0313), more distant relatives were defined as zero.
- *Coop*_*Around*_: A continuous variable that measures the cooperative context and denotes the number of gifts given or received among the other herders in the same siida as the receiver or giver. From a giver’s perspective, the variable is measured as the number of gifts given within his/her siida minus the gifts given by himself/herself. From the receiver’s perspective, the variable is measured as the number of gifts received within his/her siida minus the gifts received by himself/herself.

### Statistical Analyses

We applied multiple linear regression models (Fox, 2002; Zuur et al., 2009) in order to investigate the relationship between the size of gifts given (*Amount*_*given*_) and received (*Amount*_*received*_) by including the following model terms: (1) the main effect of the coefficient of relatedness (*r*); (2) the main effect of the level of cooperative context (i.e., the willingness of surrounding herders to cooperate; *Coop*_*Around*_); and (3) the interaction between *r* and *Coop*_*Around*_ (i.e., how an increased level of giving in the cooperative context may strengthen or weaken the effect of kinship and vice versa). The models fitted to data were thus defined a priori (meaning that our models included these estimated effects irrespective of their level of statistical significance: e.g., Anderson, 2008) as follows:

1. $${Amount}_{given}=r+{Coop}_{Around}+r\times {Coop}_{Around}$$
2. $${Amount}_{received}=r+{Coop}_{Around}+r\times {Coop}_{Around}$$

Because the amount available for the herders to give differed across regions, we ran separate analyses in the North and in the South to investigate whether there are any regional differences between the effect of kinship or the cooperative context. Visual inspection of standard diagnostic tools revealed no apparent deviations from the underlying assumptions for linear models (ESM §1). All statistical analyses and plotting of results were conducted in R (R Development Core Team, 2020), the tests were two-tailed, and the null hypothesis was rejected at an α-level of 0.05.

## Results

### Effect of Kinship

**Fig. 2:**
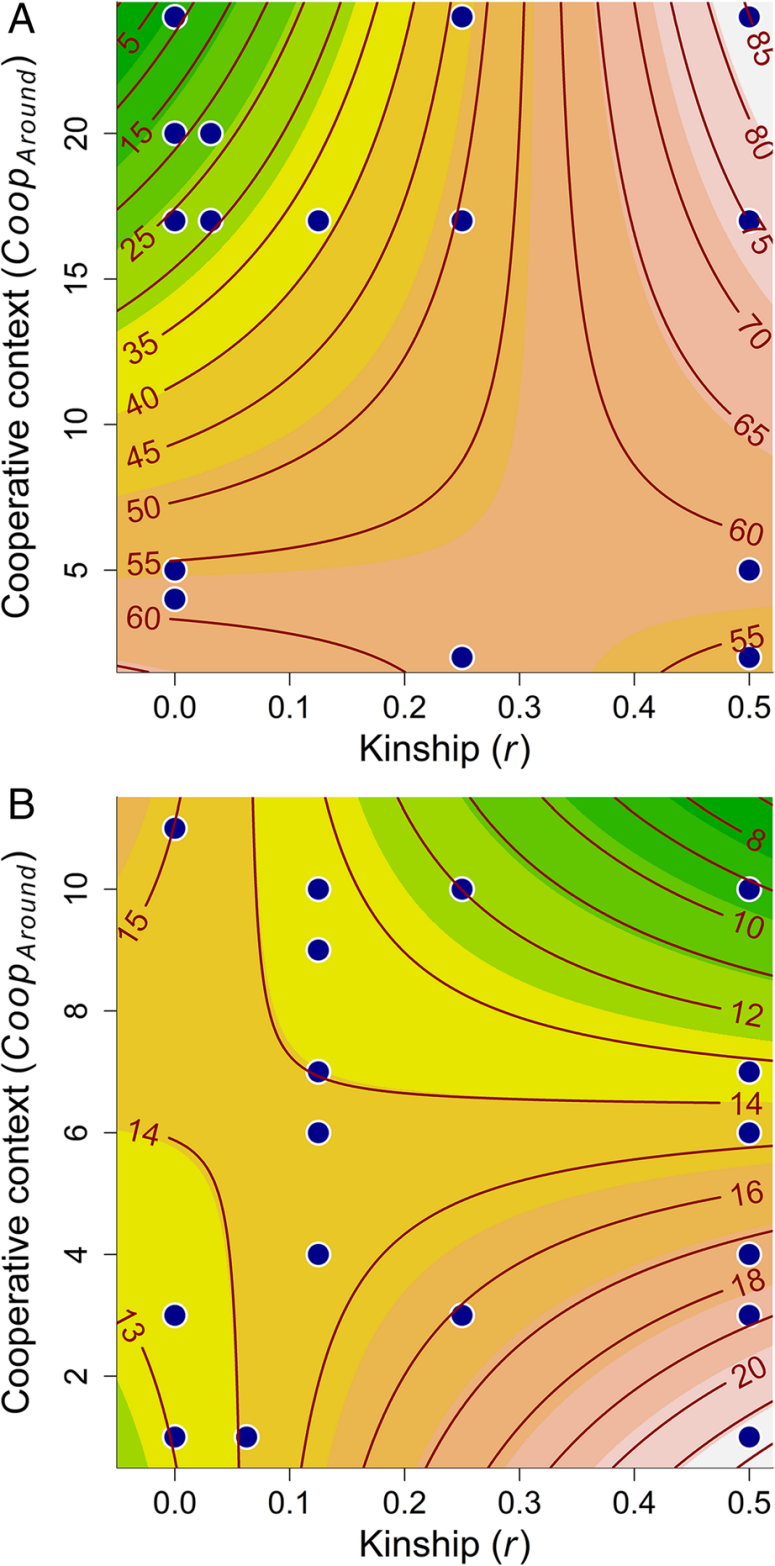
Contour plot showing the size of gifts *given* in the South (A) and North (B) as a function of the effect of the cooperative context (*Coop*_*Around*_) and kinship (*r*). Points show the scatterplot of *Coop*_*Around*_ as a function of *r*, whereas contour lines show the predicted values from the model presented in Table 3. *Please note that the predicted values are were back-transformed from log*_*e*_*- to normal-scale based on the model output presented in Table **3*. See ESM §4 for a visualization of how the two predictor variables affect the effect sizes (the estimated slope, or β) of each other

**Fig. 3:**
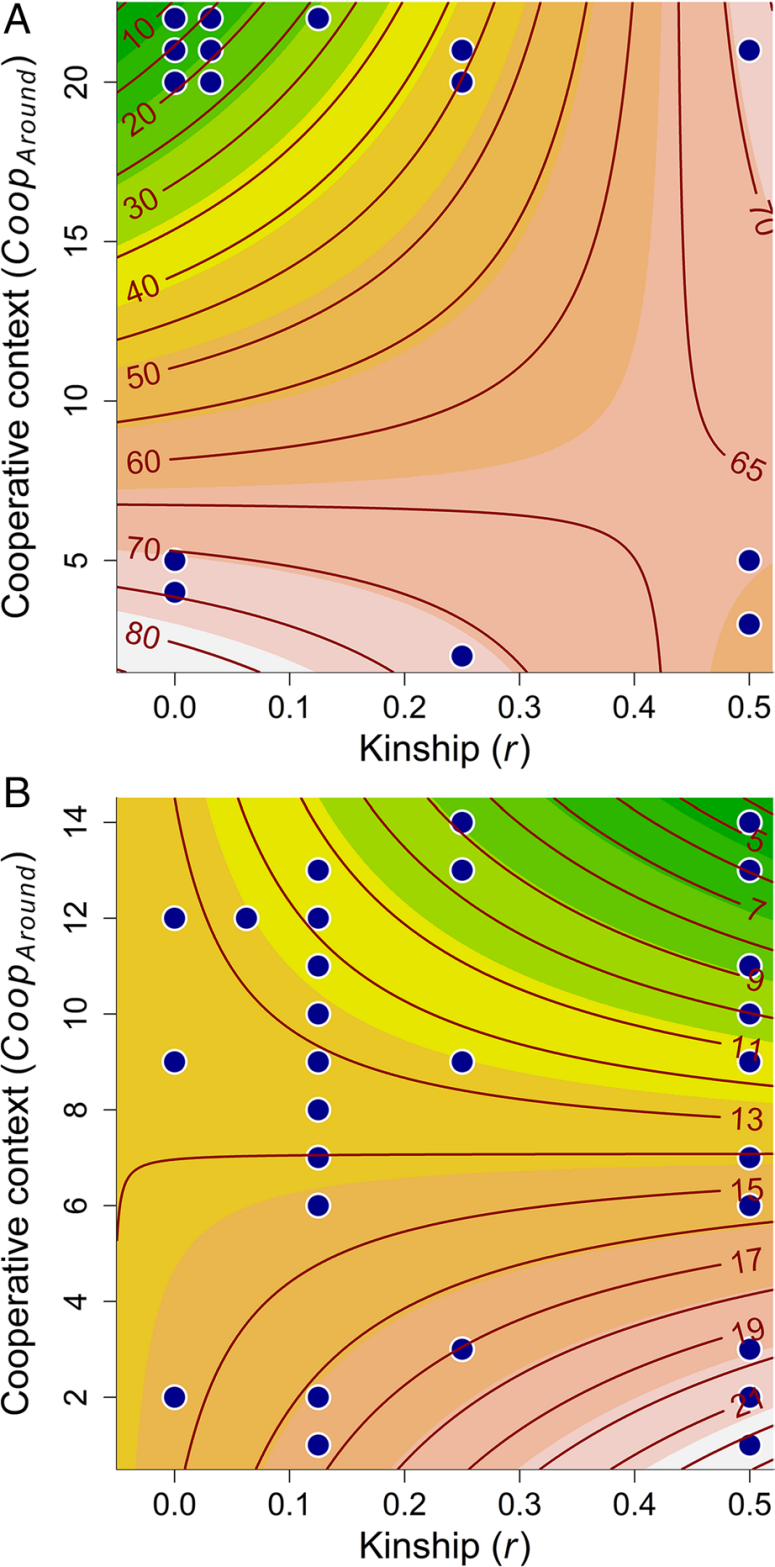
Contour plot showing the size of gifts *received* in the South (A) and North (B) as a function of the effect of the cooperative context (*Coop*_*Around*_) and kinship (*r*) from the model presented in Table 4. The caption for Fig. 2 provides technical details

In the South, the main effect of kinship (*r*) had a negative, but not a statistically significant, effect on the size of the gift, both when giving fuel (Table 3; Fig. 2A) and when receiving fuel (Table 4A; Fig. 3A). In the North, kinship had a small, but not statistically significant, positive effect on the size of the gift for both giving (Table 3B; Fig. 2B) and receiving gifts (Table 4B; Fig. 3B). In both regions, the effect of kinship was slightly larger for gifts received, relative to gifts given (see ESM §2 for scatterplots of the models).

**Table 3:** Linear models relating the size of given gifts as a continuous variable to the genetical relationship (*r*); and number of gifts given among the other members of the winter siida (*Coop*_*Around*_)

Parameter	Response: *Amount*_*given*_^a^		
	Value (95% CI)	df	*p*
Gifts given in the South^b^			
Intercept	4.074 (3.194, 4.954)	31	< .001
* r*	− 0.567 (− 3.418, 2.285)	31	.688
* Coop*_*Around*_	− 0.066 (− 0.120, − 0.012)	31	.018
* Coop*_*Around*_ × *r*	0.162 (− 0.031, 0.355)	31	.097
Gifts given in the North^c^			
Intercept	2.283 (1.600, 2.967)	52	< .001
* r*	1.196 (− 0.706, 3.099)	52	.213
* Coop*_*Around*_	0.016 (− 0.076, 0.107)	52	.737
* Coop*_*Around*_ × *r*	− 0.167 (− 0.429, 0.098)	52	.213

^a^The response variable (gifts) was log_e_-transformed

^b^One herder from the South was excluded from the analyses because he was the only herder from his siida participating in the gift game, making it impossible to calculate the *Coop*_*Around*_ variable. The results presented here are from a model with the observation excluded

^c^Four herders from the North were excluded from the analysis because they were the only herders participating in the gift game from their respective siida, and 17 were excluded due to the lack of information concerning kinship

**Table 4:** Linear models relating the size of received gifts as a continuous variable to the genetic relationship (*r*); and number of gifts received among the other members of the winter siida (*Coop*_*Around*_)

Parameter	Response: *Amount*_*received*_^a^		
	Value (95% CI)	df	*p*
Gifts received in the South^b^			
Intercept	4.588 (3.940, 5.237)	30	< .001
* r*	− 1.079 (− 3.254, 1.096)	30	.319
* Coop*_*Around*_	− 0.090 (− 0.125, − 0.056)	30	< .001
* Coop*_*Around*_ × *r*	0.164 (0.024, 0.304)	30	.023
Gifts received in the North^c^			
Intercept	2.466 (1.805, 3.126)	54	< .001
* r*	1.232 (− 0.471, 2.935)	54	.153
* Coop*_*Around*_	− 0.010 (− 0.081, 0.060)	54	.770
* Coop*_*Around*_ × *r*	− 0.154 (− 0.354, 0.046)	54	.129

^a^The response variable (gifts) is log_e_-transformed

^b^Two herders from the South were excluded from the analysis because they were the only herders who received gifts in their siida*,* making it impossible to calculate the *Coop*_*Around*_ variable. The results presented here are from a model with the observation excluded

^c^Two herders from the North were excluded from the analysis because they were the only herders that received gifts in their siida, and 17 were excluded because of a lack of information concerning kinship

### Effect of Cooperative Context

The main effect of cooperative context (*Coop*_*Around*_) was small, negative, and statistically significant on the size of the gifts given (Table 3A; Fig. 2A) and received (Table 4A; Fig. 3A) in the South. The same tendency was found in the North for gifts received (Table 4B; Fig. 3B), but this effect was not statistically significant. The main effect of cooperative context had a small and positive, but not statistically significant, effect on gifts given in the North (Table 3B; Fig. 2B; see also ESM §2 for scatterplots of the models).

### Effect of Interaction (Kinship and Cooperative Context)

There was a significant positive interaction between kinship and cooperative context on the size of the gifts received in the South (Table 4A; Fig. 3A). The sign and magnitude for this effect were the same for gifts given in the South, but this effect was only barely significant (Table 3A; Fig. 2A). The interaction effect was negative in the North (Tables 3B and 4B; Figs. 2B and 3B), but not statistically significant.

## Discussion

Overall, this study predicted that the effect of kinship and cooperative context differs regionally, with kinship being more critical in the North and the level of cooperation more critical in the South. This was partly supported since the main finding for the South was that a high level of cooperation in the South pushes the herders to distribute gifts more evenly among the other herders. At the same time, herders in the South seem to give larger gifts to close kin than to unrelated individuals. In contrast, in the North, kinship seems to be the main factor affecting gift distribution. Although herders in the North are also concerned with distributing gifts equally, this concern is limited to close kin: the cooperative context drives gifts to be distributed evenly among other closely related herders. We thus documented regional contrasts in cooperative decisions: whereas herders in the South were affected by both the cooperative context and kinship, kinship seems to be the main determinant of cooperation in the North.

### Cooperation in the South

As expected, when assessing the size of the gifts given and received, the cooperative context had a negative main effect on the size of the gifts in the South, indicating that the herders in the South both give and receive more, and smaller, gifts, rather than a few large gifts. In effect, among herders in the South, equal distribution of gifts seems to be more important than limiting it to a subset of individuals who are close relatives. In contrast, kinship seems less important: kinship had a negligible negative effect on the size of the gifts given and received in the South. This was further confirmed when herders were asked about their reasons for giving gifts: no herder in the South reported kinship as an essential factor.

The effects of kinship and cooperative context were not independent of each other. We also found a positive interaction between cooperative context and kinship in the size of the gifts received and given in the South. The effect of cooperative context seems, however, to be negative regardless of the relatedness between the giver and the receiver, except for very closely related individuals, when the effect changes to positive. Pertinently, the effect of kinship changed depending on the cooperative context: at low levels of cooperation in the group, the effect of kinship was relatively stable. In contrast, at high levels, kinship had a positive effect on the size of the gift. In short, at high levels of cooperation, herders gave larger gifts to close relatives (Fig. 2A); a similar yet less evident pattern was found for gifts received (Fig. 3A).

Overall, the results for herders in the South support Smith et al.’s (2018) finding that cooperative behavior is influenced by social context: cooperation was best predicted by the cooperativeness of an individual’s residence group. In the same way as Hazda foragers are benefiting by cooperation regardless of kinship, Saami reindeer herders in the South seem to distribute the gifts evenly among other herders. Nevertheless, although the level of intragroup cooperation drives gifts to be distributed evenly among the other herders, herders in the South still—to some degree—favor close kin as they give or receive larger gifts.

The cooperative context thus reflects levels of trust and coordination within groups that again shape herder strategies when distributing the gifts. A siida characterized by a high level of trust and willingness to cooperate, regardless of kinship, should exhibit many, small gifts given between the herders in the siida. The same factors influence the amount and size of the gifts received. That is, if a herder belongs to a siida where conditions are favorable for distributing the gifts equally among all members, the gifts received should be many and small.

In sum, the cooperative context plays an important role in explaining cooperative behavior that mirrors the history of reindeer herding in the South. If there are few conflicts among the herders, it might result in an environment with a high degree of reciprocity. Thus, herders might cooperate equally with both related and unrelated members of the siida, rather than being limited to cooperating only with kin. In effect, in the South cooperation is shaped by direct fitness benefits, and not only by indirect fitness benefits from cooperating with kin (Allen-Arave et al., 2008; Alvard, 2003; Griffin & West, 2002; Næss et al., 2012).

### Cooperation in the North

The main effect of the cooperative context in the North showed a tendency for a small positive and a negative effect on gifts given and received, respectively. In contrast, kinship had a positive effect on the size of the gifts given and received: kin gave and received fewer and larger gifts. Herders in the North also highlighted kinship as being an important factor for determining whom to give gifts to when asked about reasons for giving gifts. For instance, five of the twelve herders who gave everything to one individual reported that they did it because they were close relatives.

As in the South, the effects of kinship and cooperative context were dependent on each other. The interaction between kinship and cooperative context had a weak negative effect on the size of the gifts given and received. In contrast to the South, results for the North indicate that at low levels of cooperation in the group, kinship had a small and positive effect on the size of gifts given. At high levels of cooperation in the group, however, the effect of kinship was close to zero. Pertinently, the effect of the cooperative context changed depending on the level of kinship: at low levels of kinship, the effect of intragroup cooperation was positive whereas at high levels of kinship it was negative. In short, herders gave larger gifts to close relatives when there was a low level of cooperation. At high kinship levels, however, the size of gifts decreased as the level of cooperation increased (Fig. 2B), and a similar, yet less evident pattern was found for gifts received (Fig. 3B). Thus, while herders in the North also seem to be concerned with distributing gifts equally, this concern is limited to close kin: the level of intragroup cooperation drives gifts to be distributed evenly among other closely related herders.

The results from the North support previous findings for Saami reindeer husbandry. For instance, Næss et al. (2010) documented that the effect of labor (the number of members, or siida-shares, within a district) was dependent on kinship in order to have a positive effect on herd growth (i.e., labor interacted with kin analogous to the way cooperative context was dependent on kinship in our study). Cooperative labor investment was thus mediated by kin relations in Næss et al.’s (2010) study: it was not sufficient to have access to a potentially large workforce if there was a lack of coordination, nor was it sufficient to have a well-coordinated group if there were too few people to accomplish the necessary tasks.

In sum, kinship plays an important role in explaining cooperative behavior that mirrors the history of reindeer herding in the North, where the history of herder-herder competition has exacerbated herder conflict and resulted in a lack of trust between herders (for details, see Næss, 2020). Thus, in a social environment characterized by conflicts, kinship might be a safe bet concerning cooperation.

## Assumptions and Limitations

### Seasonal Aspects of Cooperation

There might be several explanations for the difference in the results between North and South. For example, in the North, the explicit aim was to interview herders utilizing winter pasture areas managed in common whereas in the South, the aim was to cover all the summer districts. Importantly, compared with the South, herding in the North is organized differently in winter and summer. In the North, there are seasonal differences in siida composition: winter siidas are smaller than summer siidas and may not even be composed of the same people (Næss, 2020). In effect, herders in the North do not necessarily cooperate with the same individuals year-round. In contrast, in the South, there is no seasonal difference in siida composition: they cooperate with the same individuals throughout the seasons. This regional difference might facilitate a more stable cooperative environment in the South compared with the North. For example, Hausner et al. (2012) found a low level of trust and cooperation in winter pastures in the North; 52% of the respondents (*n* = 74) were suspicious of their neighbors. Only 19% reported a substantial degree of trust toward neighboring herdsmen (Hausner et al., 2012). In contrast, during summer, trust is high: most summer pastures are managed by one siida, whose members have strong family ties with a long history of collaboration (Hausner et al., 2012). In effect, in the North, the cooperative aspect of herding might differ between winter and summer, with kinship being more important during winter and the cooperative context more important during summer. This difference is not necessarily captured in this study, and the seasonal reshuffling of group membership and its possible effect on cooperation is something that needs to be considered in future studies.

### The Importance of Siida Membership

Furthermore, Thomas et al. (2015) found that belonging to the same siida was a strong predictor of the recipients of herders’ gifts. Similarly, both herders in the North and the South often reported that they gave fuel to a given person because they belonged to the same siida as themselves. In effect, not controlling for gifts distributed within siidas might impact our conclusion in several ways. First, siidas in the South consist of five to ten license owners whereas in the North, the winter siidas are smaller, consisting of two to seven licenses. Thus, when deciding to distribute gifts among siida members, herders in the North have fewer possible recipients if deciding to give gifts to members of one’s own siida. In contrast, herders in the South can distribute the gifts to more people and still only give to members of the same siida (see below).^4^

Pertinently, giving a gift and the amount to be given might entail different decisions. While the probability of giving a gift increased with membership in the same siida (Thomas et al., 2015), it is not apparent that the same should be the case concerning the size of the gift. In a probabilistic context, gifts of 5, 35, or 100 L are all equal. Sizes of gifts are, however, not equal. As the results from the South indicate, herders simultaneously aim to distribute gifts evenly among the other herders and favor close kin, to whom they give or from whom they receive a larger gift than others. In effect, siida membership might be an essential factor when deciding on the recipient but may have little effect on the amount herders give to each other. This is not to say that siida membership is unimportant. It is, since only one of 36 (2.7%) and 12 of 72 (16.7%) gifts were distributed to individuals from another siida in the South and North, respectively. In effect, gifts are predominately distributed within siidas. Nevertheless, since including membership in the same siida as a covariate in the analyses did not impact our inferences (results not shown), we chose to exclude it in our analyses.

### Differences in Study Design

Another important aspect concerns the total amount of fuel available for the herders in the North (35 L) and the South (100 L) to give away. Since herders in the South had almost three times as much petrol to give away, they could more easily distribute many gifts or one large gift compared with the herders in the North. Rather than measuring differences in cooperation between the North and South—for example, that the high level of intragroup cooperation in the South pushed the herders to distribute gifts more evenly—perhaps our findings are simply an artifice of differences in study design. In short, herders in the North did not have the same option because they were limited by the amount available for them to distribute and therefore they could give fewer gifts compared with herders in the South. Regardless of the limit placed on the gifts, the decisions are structurally similar: a herder could choose to give one relatively large gift or many relatively small gifts in both regions. Moreover, the results reflect this: although herders in the South were concerned with distributing gifts evenly, they distributed larger gifts to close kin than to unrelated individuals. And although herders in the North distributed the largest gift to close relatives, they were also concerned with distributing gifts evenly among this group of closely related herders.

Furthermore, the median number of gifts given in the South was 1, and the maximum was 8 (mean = 2.4). This is quite similar to the North: the median number of gifts was 2 and the maximum was 7 (mean = 2.7). Thus, the gift-giving decisions seem not to be impacted by differences in study design (for details of gift distributions, see ESM §3).

### Confounding and the Problem of Autocorrelation

Observational studies have potential problems concerning confounding that may lead to spurious relationships between predictors and response as well as biased estimates (Cohen et al., 2003). We reduced this potential by including a priori expectations for all predictors in the analyses (Anderson, 2008; Burnham & Anderson, 2002). One critical factor concerning gift-giving might be the age of the recipient. Several herders in both the North and the South stated that they would like to help younger herders and therefore gave gifts to them. Six of the gifts in the North and three in the South were given because the recipient was young or had recently got their license. However, since a previous study found that age had no significant effect on the number of gifts received (Thomas et al., 2015), we chose to exclude it from our analyses.

An argument could also be made that *group size* (the number of herders in a siida) is a vital confounder for gift-giving and receiving. However, the size of the siida and *Coop*_*Around*_ was correlated (ESM §1). High or even moderate collinearity is problematic when effects are weak (as in this study). It may result in nonsignificant parameter estimates (i.e., the precision of the estimates will decrease; see Licht, 1995) relative to a situation without collinearity. With collinearity removed, variables may become significant, indicating that collinearity problems may render significant terms nonsignificant (Zuur et al., 2009). More to the point, if collinearity is ignored, statistical analysis might indicate that nothing is significant, but dropping one predictor could make others significant or even change the sign of estimated parameters (Zuur et al., 2009).^5^

In short, we were left with a choice of whether to include the predictors for which we had a priori expectations (from a theoretical point of view) or to replace them with other possible essential covariates. From a statistical point of view, such problems fall under the purview of specification error. In this case, it could be argued that the problem is mainly related to estimating a model with the wrong set of predictors. Pertinently, decisions regarding which predictors to include or exclude cannot be assessed statistically but must be based on theoretical considerations relevant to the hypotheses tested (Berry & Feldman, 1985; Licht, 1995). Consequently, we chose to focus on *Coop*_*Around*_ because this was the variable we had both a theoretical interest in and a priori expectations for (see Fox, 1991, p. 15 for a similar argument). Moreover, we argue that *Coop*_*Around*_ represents a novel theoretical concept that to some degree incorporates and controls for siida size.

Another issue concerns a possible violation of independence due to the nature of the data used in this study (Zuur et al., 2009). In short, if a herder decides to give two gifts, the size (or amount) of the second gift will be dependent on the size of the first gift since each person has a fixed amount of petrol to give away. The amount received is, however, less dependent on what a person has previously received. A possible solution is to fit Linear Mixed Effects (LME) models with individuals as random effects (e.g., Pinheiro & Bates, 2000; Zuur et al., 2009). Such an approach might account for a lack of independence by estimating, and accounting for, within- and among-person variability either in the intercept (constant term) or in one or more slopes (either kin or intragroup cooperation in this study; see, e.g., Harrison et al., 2018). This was not a feasible solution since the number of repeated measures per person was low in our analyses: ≥ 50% of the respondents gave only one or two gifts (ESM §3). Nevertheless, we performed two additional analyses to assess issues concerning repeated measures by (1) refitting models to aggregated data using person-specific averages for both the responses and the predictors in question and (2) refitting the models with the sum of weights per person set to one (e.g., an observation for a person giving *one* gift received a weight of *one*, whereas the observations for a person giving *four* gifts received a weight of *0.25* each). Since these attempts to account for problems concerning repeated measures did not change our conclusions, we chose to fit regular linear models to our data as this seems to represent a simple yet robust statistical approach for dealing with our data.

### Framing Effects and the Cultural Salience of Using Petrol as a Currency

Experimental economic games have proven to be well suited for eliciting cooperative behavior (Thomas et al., 2015, 2016, 2018). By using fuel as gifts, the reindeer herders distribute a value that has an impact on their daily lives; petrol is a commodity used by reindeer herders for day-to-day tasks and large-scale seasonal migrations involving intensive periods of collaboration. Moreover, distributing gifts that would benefit the receiver is relevant for the reindeer herders since reindeer husbandry is dependent on collaboration and collective actions (Næss et al., 2009, 2010, 2012; Thomas et al., 2015, 2016, 2018).

Nevertheless, *framing* might significantly affect decisions: for example, the same information can lead to different decisions depending on the way that information is interpreted (Levin & Lauriola, 2003). In experimental games among the Maasai, framing a trust game as *osutua*—gift-giving relationships based on obligation, need, respect, and restraint—shifted the gameplay away from the logic of investment toward the mutual obligation of the partners: in fact, it reduced transfers of money between players compared with games with no framing (Cronk, 2007). Framing effects have also been noted in Kenya, where pastoralists contributed generously in a public goods game because participants identified it as a version of a village-level public goods situation such as building a school (Henrich, 2004). In effect, this demonstrates both framing and spillover effects (Peysakhovich & Rand, 2016), whereby the rules of the game in one setting can strongly influence behavior in other unrelated settings.

Although petrol is essential in the daily lives of reindeer herders, little is known as to the degree of actual petrol sharing among herders. In effect, the gift game was not framed in terms of a known shared commodity but rather concerning a currency that is important for herders and easily used in a game setting. Nevertheless, the game was structured around a known ethos or norm of sharing or helping other members of the same siida (Næss et al., 2021; Paine, 1970). In the North, most herders report that they borrow equipment from others and that everyone helps each other. In the South, answers were a bit different: herders stressed that since everyone has what they need, little borrowing occurs. Nevertheless, there is an aspect of sharing among herders since the siida owns some equipment that can then be used by members. Further, if equipment gets broken, herders can borrow from neighbors, and if someone needs equipment that not everyone has access to (e.g., a car trailer), herders can borrow from individuals who do have it. Concerning help, the family was considered the most important factor in the South. There are no reasons to expect that the sharing of petrol should follow a different pattern.

In contrast to other experimental games, the game herders played here was only partly anonymous: participants were given an amount of fuel to distribute among other licensed herders known by name and decided to whom and how much they would give. The identity of the giver was only unknown to the receiver (the only restriction being that the gift had to be given to another licensed herder within the region). Because the gifting events occurred over several months, it is possible that at least some of the observed behavior was due to participants arranging, outside of the game, to give gifts to one another (Gervais, 2017).

### Cost-free Cooperation?

Gift-giving in the economic games used in this study might arguably be unsuitable as a proxy for cooperation, as defined by some evolutionary scientists (e.g., Bowles & Gintis, 2003; Fotouhi et al., 2018; Nowak, 2006), since herders in our study were prohibited from keeping fuel for themselves. Consequently, distributing fuel carried no cost, and as such, gift-giving might not measure cooperation correctly. Smith et al., (2019, p. 84) argues, for example, that while the gift-giving game, as played with reindeer herders, “allows the choice of giving to multiple individuals, it does not measure levels of cooperation as there is no option for keeping gifts for one’s self, meaning that there is no conflict between individual and group interests.”

That, however, is arguably a restricted view of cooperation. Cooperation might not always entail costs; the simplest form of cooperation entails some form of coordination, such that when individuals share preferences, cooperation is always beneficial (see, e.g., Smith, 2003). Alvard and Nolin (2002) argue, for example, that many situations of human cooperation are better represented by mutualism (see also Richerson et al., 2003; West et al., 2007).^6^ This is not to suggest that the gift games used in this study are an example of cooperation as coordination (although we cannot exclude the possibility that participants coordinated, outside of the game, to give gifts to one another; see above). Rather, we suggest that while this type of gift-giving is cost-free, it is an important measure of friendship, or of whom you want to cooperate with, and has thus been argued to be a valid measure of social connections (e.g., Glowacki et al., 2016). Thus, gift-giving effectively reveals information about existing social relationships, allowing some insight into the patterns of cooperation among Saami reindeer herders (Thomas et al., 2018; see “Methods” section, above).

### Gifts Given vs. Gifts Received

Mysterud et al. (2006) found that kinship was an important predictor for gift-giving at Christmas among Norwegian students: more money was spent on gifts for close kin. Importantly, although the number of people to whom the students gave gifts was similar to the number of people they received gifts from, the individuals were not always the same: 48% gave to one or more people without receiving a gift back, and 42% received gifts from one or more people without giving back. In effect, there is a difference in giving and receiving gifts (the two might in fact be argued to be the opposite of each other) even though a single gift is the same irrespective of perspective. A giver makes an active choice, whereas the receiver’s only options are to accept or decline. Nevertheless, in this study, a single gift has the same size (in liters) whether it is considered from a giver’s or a receiver’s point of view. Thus, the amount of petrol in a single gift is identical for the two analyses. Similarly, the coefficient of relatedness, like the size of the gift, is the same regardless of whether we take the giver’s or receiver’s perspective. The main difference concerns how we have operationalized the level of cooperation around an individual. From a giver’s point of view, the cooperative context was measured as the number of gifts given within the focal individual’s siida minus the gifts given by the focal individual. In effect, it measures the willingness of siida members to give gifts or the level of cooperation in a siida. More to the point, it measures the actions undertaken by neighboring herders and how they affect the actions of the focal individual. The actions of neighboring herders have been found to be an important predictor for individual herders’ slaughter strategies: they are influenced not only by herd size but also by the amount of slaughter undertaken by neighboring herders (Næss et al., 2012). Similarly, this study shows that the size of gifts given is not only influenced by the focal individual’s own state (kinship) but also by the actions of neighboring herders (i.e., the level of intragroup cooperation). In contrast, from a receiver’s point of view the cooperative context was measured as the number of gifts received within the focal individual’s siida minus the gifts received by himself/herself. Thus, this measure is not a measure of the actions of neighboring herders to the same degree as from the giver’s point of view. Nevertheless, it measures the flow of gifts around an individual and thus provides valuable information about the cooperative behavior in a group. Moreover, giving a gift does not guarantee a participant will receive a gift and vice versa, or that the giver and receiver belong to the same siida. Thus, including the receiver’s perspective gives broader insight into the social structure than what would be the case if only gifts given were considered. Nevertheless, possible differences in giving and receiving gifts require further investigation.

## Concluding Remarks

Reindeer herding in the northern parts of Norway has a history of between-herder competition exacerbating conflict, lack of trust, and subsequent coordination problems. In contrast, due to herder-farmer competition, the southern parts are characterized by high levels of coordination and trust among herders. Consequently, we expected that herders in the South are more influenced by a principle of equality—favoring everyone in the group—rather than nepotism—when distributing gifts. In contrast, the opposite was expected from herders in the North. This was only partially supported: among herders in the South, equal distribution of gifts seems to be more important than limiting it to a subset of individuals who are close relatives.

Nevertheless, herders in the South do favor kin to some degree. Although the level of intragroup cooperation pushes gifts to be distributed evenly among other herders, close kin still give or receive larger gifts than others. In contrast, in the North, kinship seems to be the main factor affecting gift distribution. Herders in the North are also concerned about distributing gifts equally, but this concern is limited to close kin: the cooperative context drives gifts to be distributed evenly among other closely related herders.

In general, social dilemmas are ultimately about cooperation, or, more pertinently, the lack thereof. Although the act of cooperating results in the best collective outcome, it is not always clear whether it will yield the best individual outcome (Fotouhi et al., 2018). Globally, we face social dilemmas such as climate change, mass extinction of the largest animals and plants, increasing air pollution, antimicrobial resistance, emerging and re-emerging infectious diseases (e.g., Ebola, Zika, influenza, and the ongoing COVID-19 pandemic), and overexploitation of renewable resources (Bloom & Cadarette, 2019; Enquist et al., 2020; Travis, 2003). Just as we have an increasing need for more theoretically sophisticated discourses on human cooperation, the anthropological discourse on cooperation continues to be dominated by use of forager societies as an analogy for human evolution.

In contrast, less attention has been given to cooperation among nomadic pastoralists. Although they arrived later on the scene, cooperation was just as important—if not more so—for nomadic pastoralists. With the domestication and herding of livestock, coordination and cooperation would have been necessary for protecting both the herds and fellow humans from other humans and predators, as well as the efficient utilization of domesticated livestock. Furthermore, nomadic pastoralists have a historically demonstrated ability for both small-scale and large-scale cooperation (Turchin, 2007). Extending our understanding of cooperation to also include pastoralists is thus an important building block for solving pressing collective action problems.

## Supplementary Information

Below is the link to the electronic supplementary material.

Supplementary file1 (PDF 691 kb)

## Data Availability

In order to protect the privacy of participants, the data used in this study are not publicly deposited, however, the data that support the findings are available from the authors upon request.

## Footnotes

1 In contrast, the five largest winter pastures encompass 11–21 siidas, whereas the five smallest winter pastures are managed by one to six siidas each (Hausner et al., 2012).

2 Previous studies on reindeer husbandry in northern Norway have been conducted in West- and East-Finnmark; the southern areas have included North-Trøndelag, South-Trøndelag, and Hedmark. The present study only includes West-Finnmark and Troms in the North and South-Trøndelag and Hedmark in the South.

3 In October 2019, 1 L of petrol cost approximately NOK 18 (2.04 USD).

4 However, no such limitations were placed on the distribution of gifts: herders were not required to distribute to individuals in winter (vs. summer) siidas or within the same siida, but rather to individuals having a siida-share anywhere in the (North vs. South) region. Nevertheless, herders might have taken as their starting point that gifts should be given to individuals with siida-shares in winter siidas.

5 Note, however, that since collinearity is present to some degree in all observational studies, the most reasonable course when facing it may be to recognize it and live with the consequences (Berry and Feldman 1985, p. 49).

6 Although “mutualism” is usually used by ecologists with reference to cooperation between species, the meaning here is that of “mutual benefit”: a behavior that is beneficial to both the actor and the recipient (West et al., 2007).

## Code Availability

The R code that supports the findings of this study is available from the authors on request.
