# Supplementary material for: The Relative Importance of “Cooperative Context” and Kinship in Structuring Cooperative Behavior: A Comparative Study of Saami Reindeer Herders
Source: Hum Nat. 2021 Oct 20;32(4):677–705. doi: 10.1007/s12110-021-09416-6 (PMC8526998; doi:10.1007/s12110-021-09416-6)
Supplement: Supplementary file 1 — Supplementary file1 (PDF 691 kb) [file 12110_2021_9416_MOESM1_ESM.pdf]

Electronic Supplementary Material (ESM)

for

The Relative Importance of “Cooperative Context” and Kinship in Structuring Cooperative Behavior: A Comparative Study of Saami Reindeer Herders

Guro Lovise Hole Fisktjønmo, Marius Warg Næss, and Bård-Jørgen Bårdsen

Human Nature 32(3), 2021. https://doi.org/10.1007/s12110-021-09416-6

Contents

Section 1: Collinearity 2
Section 2: Scatter plot 4
Section 3: Gift Distribution 5
Section 4: Interaction plot 6

Section 1: Collinearity

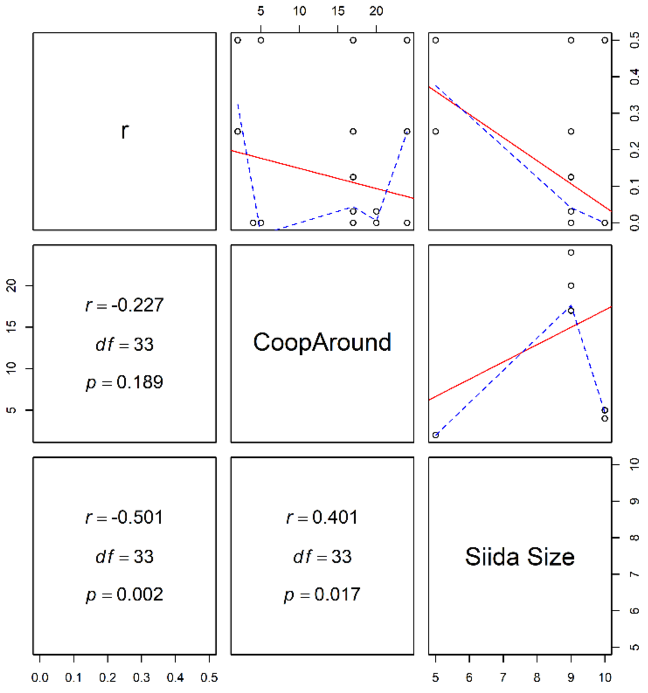

Fig. 1.1 Pairplot shows the variables used in the linear model for gifts given among herders in the South. The plots are made using the "pairs" function with default settings in R (R Development Core Team, 2020) on the linear model from the study. The pairplot revealed no clear deviations from the underlying assumptions for linear models.

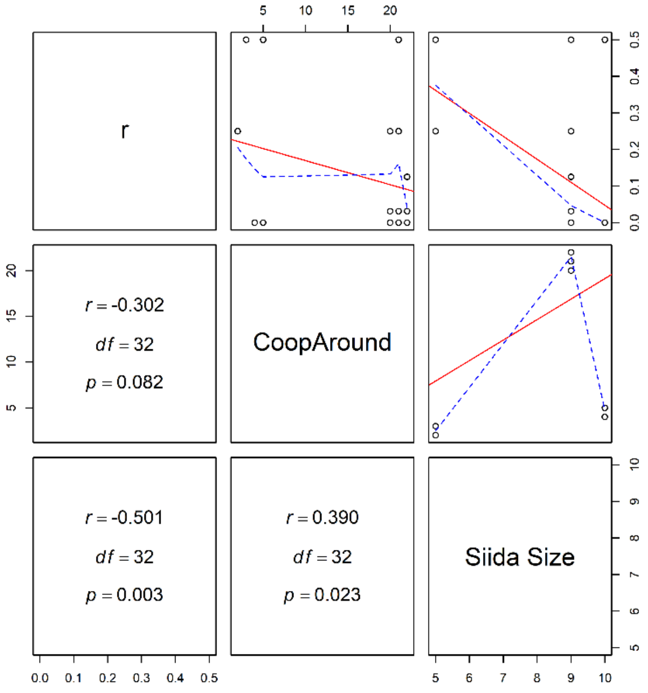

Fig. 1.2 Pairplot shows the variables used in the linear model for gifts received in the South (for technical details, see Fig. 1.1).

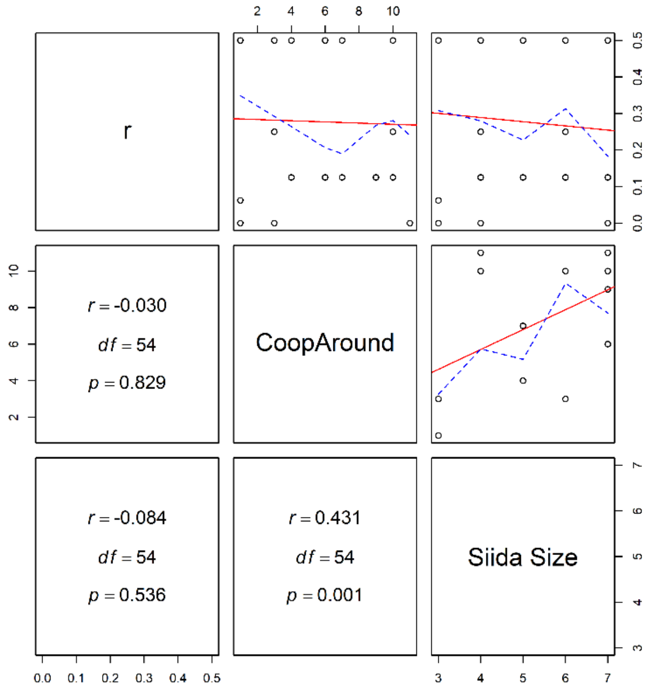

Fig. 1.3 Pairplot shows the variables used in the linear model for gifts given in the North (for technical details, see Fig. 1.1).

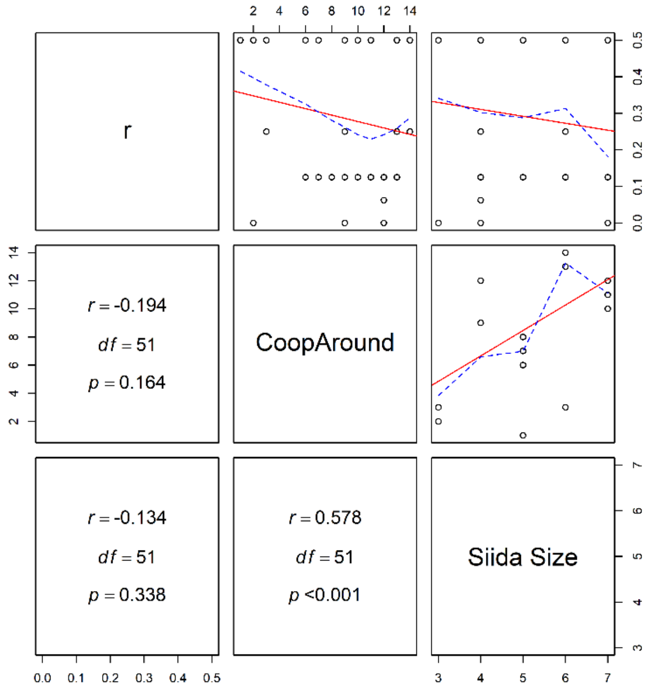

Fig. 1.4 Pairplot shows the variables used in the linear model for gifts received in the North (for technical details, see fig. 1.1).

Reference

R Development Core Team. (2020). R: A language and environment for statistical computing. Vienna, Austria: R Foundation for Statistical Computing.

Section 2: Scatter plot

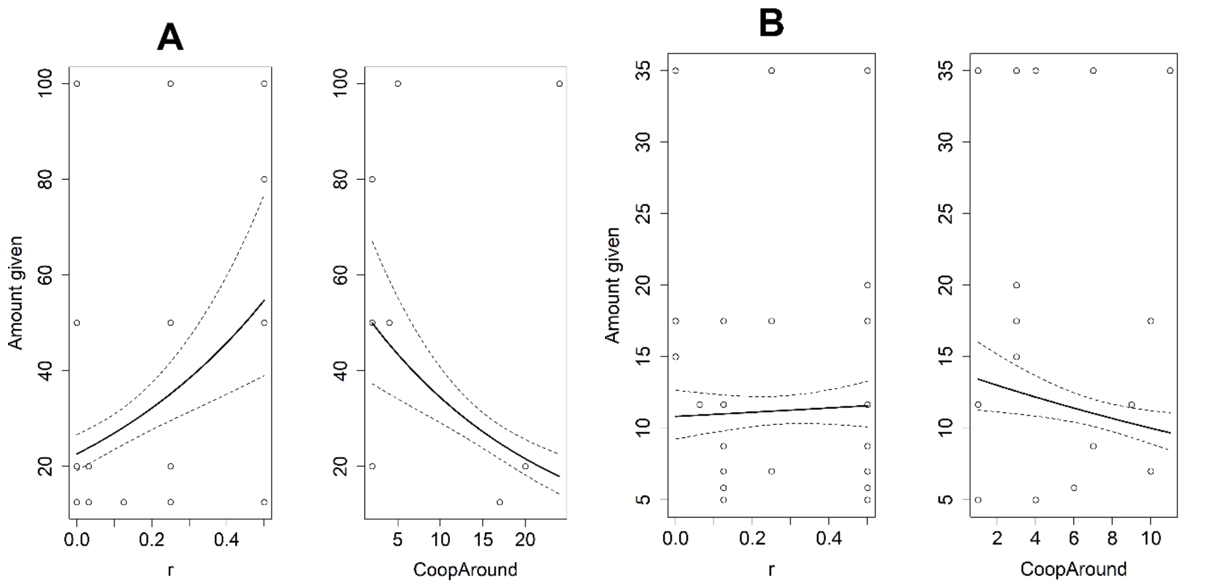

Fig. 2.1 Scatter plot showing the size of the gifts given in litres of fuel ($Amount_{given}$) as a function of kin (*r*) and the number of gifts given among the other herders in the participants' winter *siida* ($Coop_{Around}$) for A) herders in the South, and B) herders in the North. The values are back-transformed from log_e- to normal-scale from the model estimates presented in Table 3, with the hatched lines showing the predicted values (± 1 Standard Error).

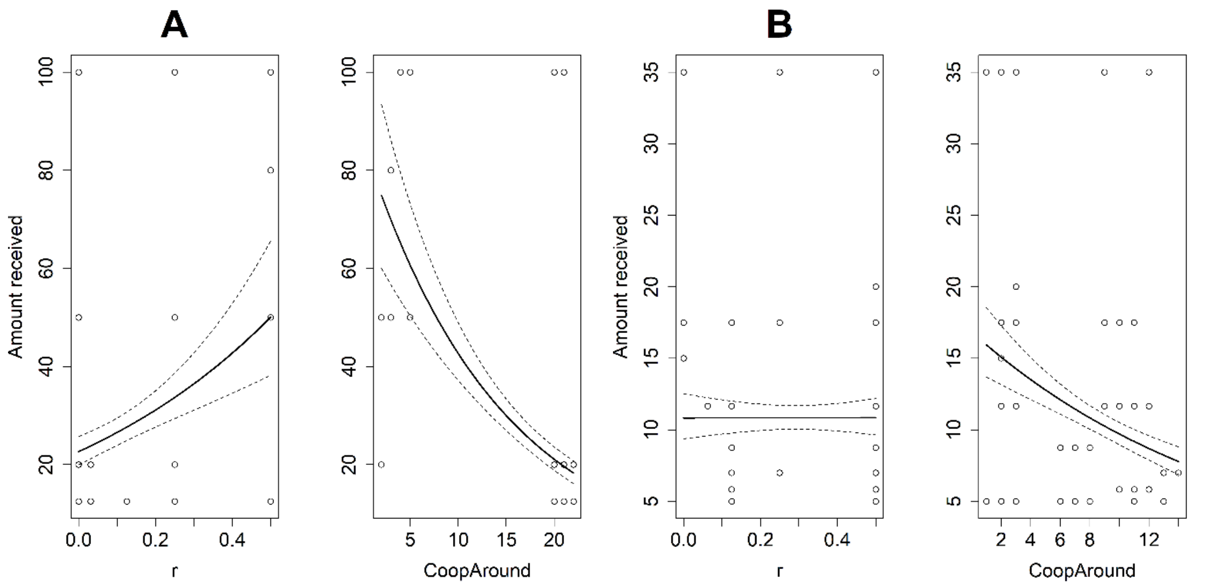

Fig. 2.2 Scatter plot showing the size of the gifts received in litres of fuel ($Amount_{received}$) as a function of kin (*r*) and the number of gifts received among the other herders in the participants' winter *siida* ($Coop_{Around}$) for A) herders in the South, and B) herders in the North. The values are back-transformed from log_e- to normal-scale from the model estimates presented in Table 4, with the hatched lines showing the predicted values (± 1 Standard Error).

Section 3: Gift Distribution

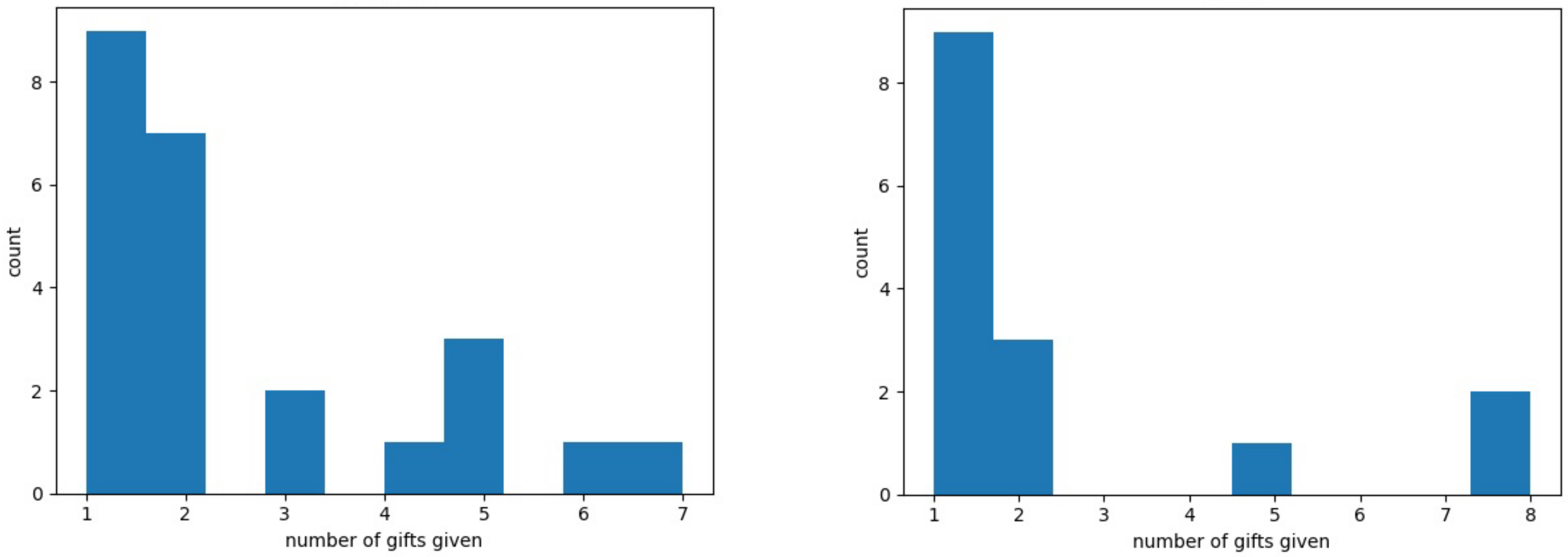

Fig. 3.1. Showing the distribution of gifts for the North (left) and South (right).

Section 4: Interaction plot

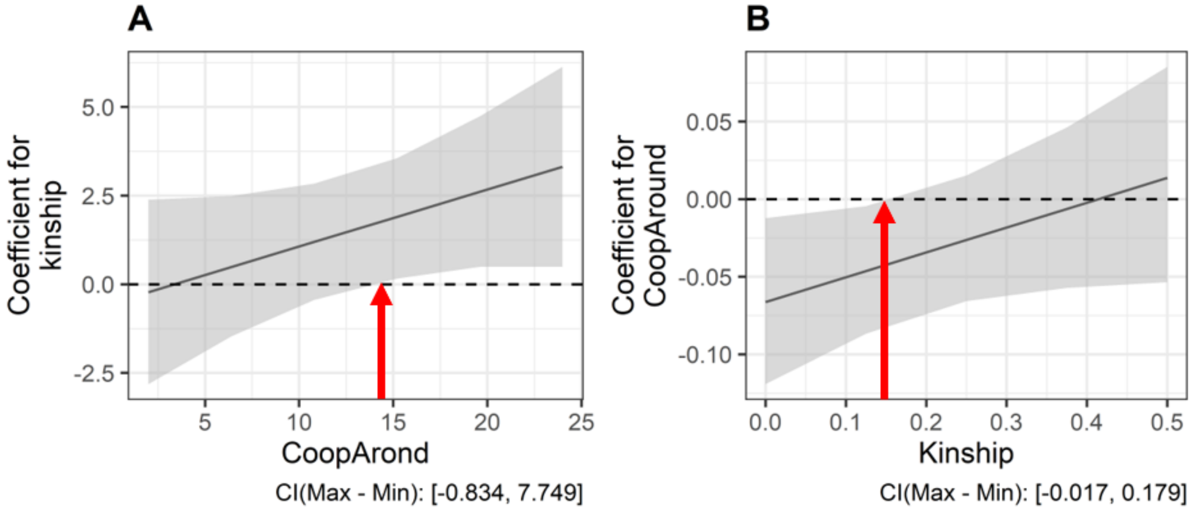

Fig. 4.1 Interaction plot shows conditional coefficients size of gifts given (*Amount*$_{given}$) among reindeer herders in the South. These interaction plots illustrate that the kinship and cooperative context need to be interpreted in light of each other, as the effect of one variable is dependent on the level of the other. The plots are made using the package "interplot" with default settings in R (R Development Core Team, 2020) on the linear model from the study. The red arrow indicates the value of one predictor where the coefficient of the other predictor becomes significant. The caption at the right bottom corner shows respectively the confidence intervals of the difference between the conditioned effects of kinship or cooperative context at the minimum and maximum values of cooperative context or kinship. The plots shows (A) the effect cooperative context (*Coop*$_{Around}$) have on the coefficient of kinship (*r*); and (B) the effect kinship (*r*) has on the coefficient of cooperative context (*Coop*$_{Around.}$).

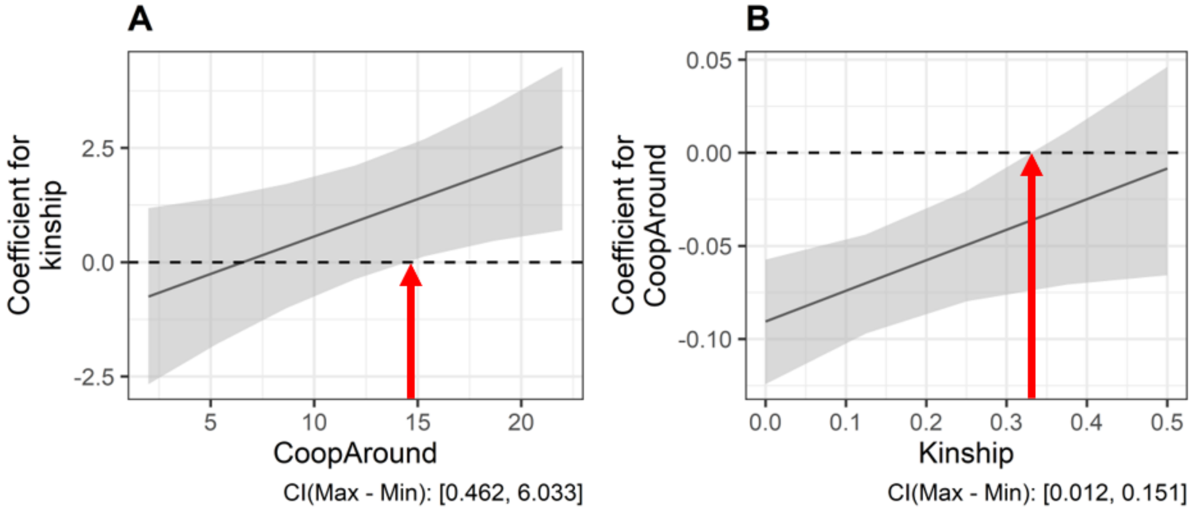

Fig. 4.2 Interaction plot of how the variables affect each other's effect on the size of gifts received (*Amount*$_{received}$) among reindeer herders in the South. The plot shows (A) the effect cooperative context (*Coop*$_{Around}$) have on the coefficient of kinship (*r*); and (B) the effect kinship (*r*) have on the coefficient of cooperative context (*Coop*$_{Around.}$) (for technical details, see Fig. 4.1).

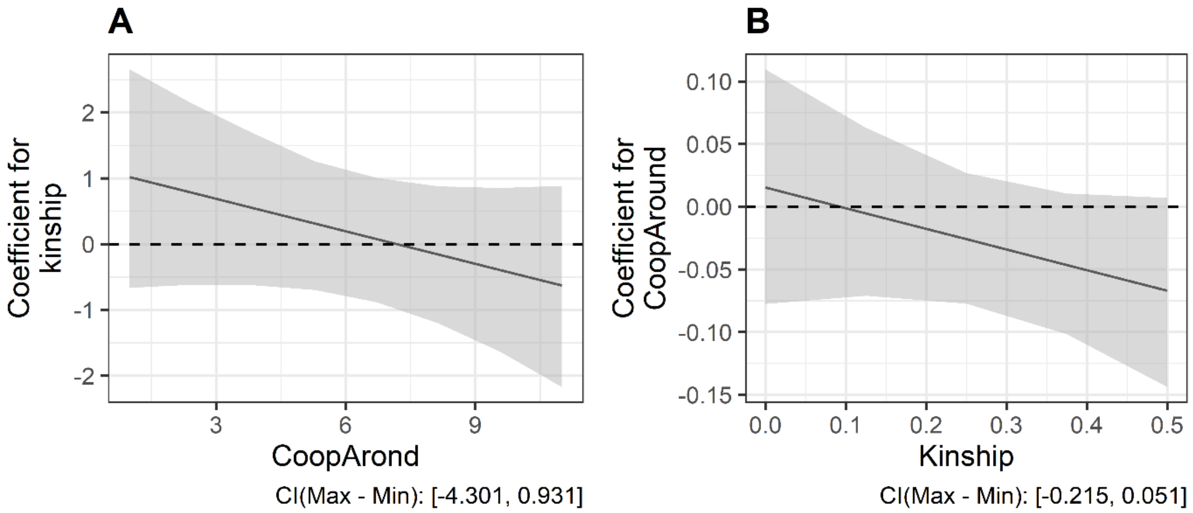

Fig. 4.3 Interaction plot of how the variables affect each other's effect on the size of gifts given ($Amount_{given}$) among reindeer herders in the North. The plot shows (A) the effect cooperative context ($Coop_{Around}$) have on the coefficient of kinship (*r*); and (B) the effect kinship (*r*) have on the coefficient of cooperative context ($Coop_{Around.}$) (for technical details, see Fig. 4.1).

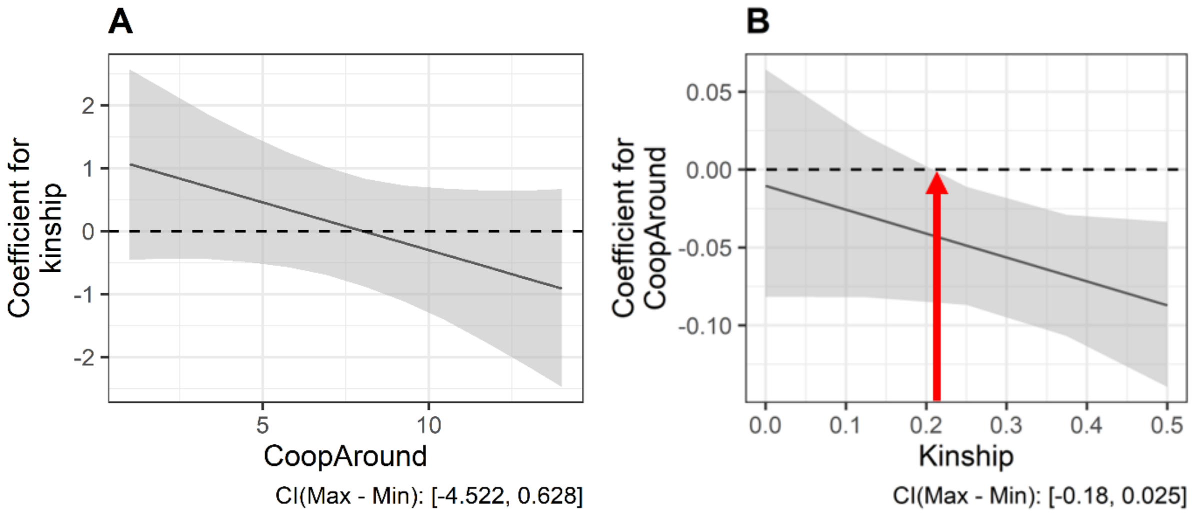

Fig. 4.4 Interaction plot of how the variables affect each other's effect on the size of gifts received ($Amount_{received}$) among reindeer herders in the North. The plot shows (A) the effect cooperative context ($Coop_{Around}$) have on the coefficient of kinship (*r*); and (B) the effect kinship (*r*) have on the coefficient of cooperative context ($Coop_{Around.}$) (for technical details, see Fig. 4.1).

Reference

R Development Core Team. (2020). R: A language and environment for statistical computing. Vienna, Austria: R Foundation for Statistical Computing.
